# Bac*Dive* in 2025: the core database for prokaryotic strain data

**DOI:** 10.1093/nar/gkae959

**Published:** 2024-10-29

**Authors:** Isabel Schober, Julia Koblitz, Joaquim Sardà Carbasse, Christian Ebeling, Marvin Leon Schmidt, Adam Podstawka, Rohit Gupta, Vinodh Ilangovan, Javad Chamanara, Jörg Overmann, Lorenz Christian Reimer

**Affiliations:** Leibniz Institute DSMZ - German Collection of Microorganisms and Cell Cultures, Braunschweig, Germany; Leibniz Institute DSMZ - German Collection of Microorganisms and Cell Cultures, Braunschweig, Germany; Leibniz Institute DSMZ - German Collection of Microorganisms and Cell Cultures, Braunschweig, Germany; Leibniz Institute DSMZ - German Collection of Microorganisms and Cell Cultures, Braunschweig, Germany; Leibniz Institute DSMZ - German Collection of Microorganisms and Cell Cultures, Braunschweig, Germany; Leibniz Institute DSMZ - German Collection of Microorganisms and Cell Cultures, Braunschweig, Germany; German National Library of Science and Technology (TIB) - Leibniz Information Centre for Science and Technology - University Library, Hannover, Germany; German National Library of Science and Technology (TIB) - Leibniz Information Centre for Science and Technology - University Library, Hannover, Germany; German National Library of Science and Technology (TIB) - Leibniz Information Centre for Science and Technology - University Library, Hannover, Germany; Leibniz Institute DSMZ - German Collection of Microorganisms and Cell Cultures, Braunschweig, Germany; Leibniz Institute DSMZ - German Collection of Microorganisms and Cell Cultures, Braunschweig, Germany

## Abstract

In 2025, the bacterial diversity database Bac*Dive* is the leading database for strain-level bacterial and archaeal information. It has been selected as an *ELIXIR Core Data Resource* as well as a *Global Core Biodata Resource*. Since its initial release more than ten years ago, Bac*Dive* (https://bacdive.dsmz.de) has grown tremendously in content and functionalities, and is a comprehensive resource covering the phenotypic diversity of prokaryotes with data on taxonomy, morphology, physiology, cultivation, and more. The current release (2023.2) contains 2.6 million data points on 97 334 strains, reflecting an increase by 52% since the previous publication in 2021. This remarkable growth can largely be attributed to the integration of the world-wide largest collection of Analytical Profile Index (API) test results, which are now fully integrated into the database and searchable. A novel Bac*Dive* knowledge graph provides powerful search options through a SPARQL endpoint, including the possibility for federated searches across multiple data sources. The high-quality data provided by Bac*Dive* is increasingly being used for the training of artificial intelligence models and resulting genome-based predictions with high confidence are now used to fill content gaps in the database.

## Graphical Abstract

**Graphical Abstract:**
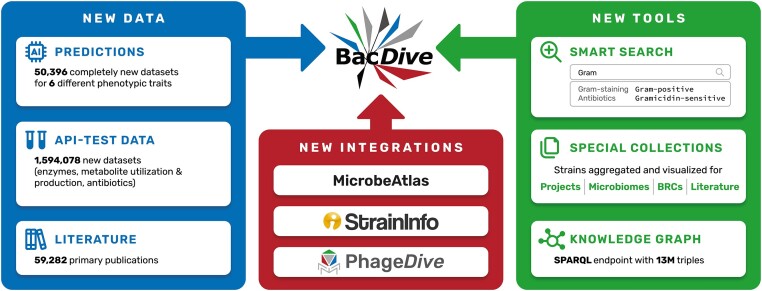

## Introduction

Bac*Dive* is the largest database for standardized information on prokaryotes in the world. It gathers and standardizes phenotypic strain-level research data from diverse sources including internal catalogs of culture collections and primary literature to make them easily accessible. These efforts were recently recognized by the Global Biodata Coalition and the ELIXIR European life science infrastructure, who both awarded Bac*Dive* the distinction of a *Core Data Resource*.

A look at recent publications using Bac*Dive* data gives a good overview on the broad research community that relies on this resource. Applications range from biotechnology, using Bac*Dive* to find bacteria for the degradation of toxic waste (1) or plastics (2) to the medical research identifying bacteria linked to oral health (3). They also include research investigating the evolutionary metabolic adaptation of specific genera (4) as well as plant health, benefiting from a group of plant-associated flavobacteria (5). A growing trend that can be identified is the use of large data sets in bioinformatic analyses and tools. Datasets that serve specific research applications are developed with the help of Bac*Dive* data, like Omnicrobe (6) focusing on habitat information, or MariClus, a dataset dedicated to marine natural products (7). Very exciting are those approaches that take advantage of the great potential of the extensive, standardized data in Bac*Dive* to make predictions. TemBerture (8) has predicted protein thermostability based on deep learning techniques and Barnum *et al.* (9) have predicted microbial growth conditions based on amino acid composition. Surely, well-designed models relying on high-quality data have the potential to close the gaps of knowledge for the already known microbial diversity and to improve the understanding of the so far unknown microbial dark matter. Here, we describe the recent developments in the *Core data resource* Bac*Dive* and how we utilize genome-based models to significantly improve the knowledge about prokaryotic strains and fill data gaps in Bac*Dive*.

## Primary content

Bac*Dive* now contains data on 97 334 strains with a total of 2.6 million data points (Release 2024.1). This represents a growth of 52% since our last report in 2021. In its current release Bac*Dive* shows data on 20 060 type strains, covering 98% of the 20 510 validly described species (https://lpsn.dsmz.de/text/numbers). The increase in type strain coverage in Bac*Dive* from just 81% in 2021 to 98% in 2024 could be achieved through improved data exchange with one of its sister databases, the *List of Prokaryotic names with Standing in Nomenclature* (LPSN) (10). Data from LPSN is no longer only used to keep Bac*Dive* up to date on correct prokaryotic nomenclature, but also to add all type strains of newly described species to the database in a timely manner. Complementing this approach, extensive data from species descriptions continue to be annotated manually and integrated into Bac*Dive*. To date, data on 7893 strains has been added from species descriptions. An overview on all strains providing manually annotated literature data is given under the special collection ‘strains from literature’ (https://bacdive.dsmz.de/collection/literature).

The database was originally conceived to make available the vast amount of data on strains collected and stored in internal files at the German Collection of Microorganisms and Cell Cultures (Leibniz Institute DSMZ). Until today, high-quality data from large culture collections forms the backbone of the database. Apart from regular updates with DSMZ data, a large data set on 13 140 strains from the Biological Resource Center of the Institut Pasteur (CRBIP, France) could recently be integrated. While only 3390 strains were completely new to Bac*Dive*, the data set significantly extended the Bac*Dive* database with comprehensive information per strain. It contained not only the public catalog data on identity, sampling, and history of the strain, but also internal observations and test results on cultivation, growth conditions, morphology, physiology and metabolism. A notable highlight is the large set of *Analytical Profile Index* (API) test results for 9783 strains.

API tests are one of the underestimated resources for systematic information about microbial strains. Developed in the 1970s, they are simple sequences of microsize physiological tests that can easily be performed in any laboratory without the need of expensive devices. Until today, these tests are routinely used in medical laboratories as well as in culture collections. In 2017, a first batch of 8977 API test results was mobilized and integrated into the Bac*Dive* database, which represented the world's largest publicly available collection of API tests at the time (11). Today the Bac*Dive* collection of API test results encompasses a total of 48 130 tests, divided into 17 test types, which provide overall 1 594 078 single data points for 24 112 strains.

**Figure 1.**
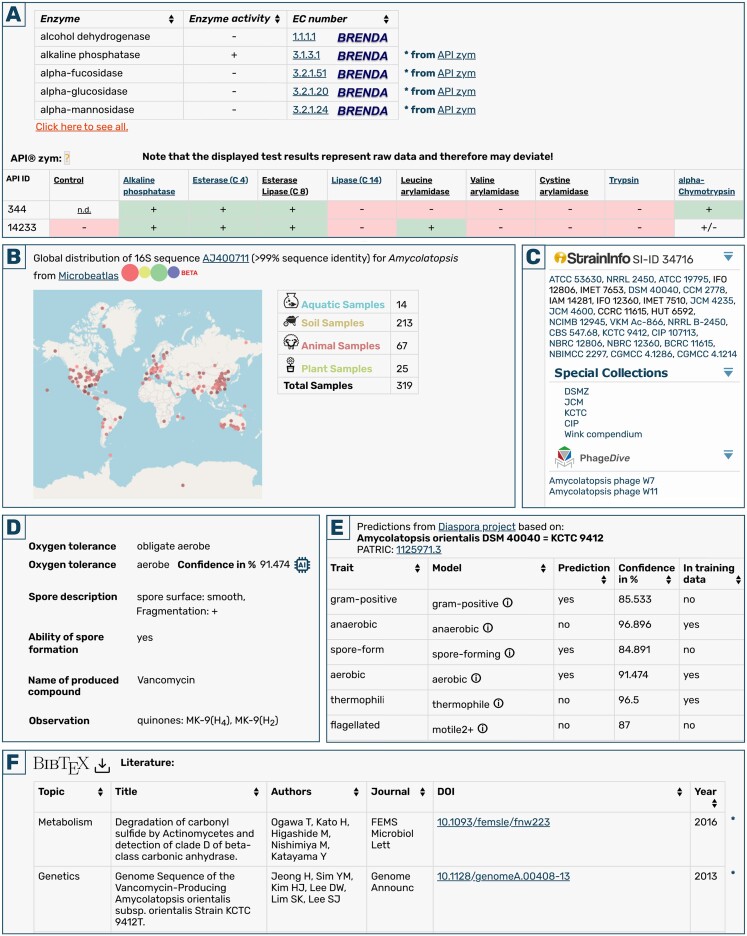
New features on the Bac*Dive* strain detail page. **(A)** Analytical Profile Index (API) test results are now not only shown in specific API tables (bottom), but also integrated alongside manually curated data. **(B)** Geographic locations and types of very closely related samples with at least 99% 16S rRNA gene sequence identity are shown as provided by Microbeatlas. **(C)** The sidebar now links to related StrainInfo and Phage*Dive* entries as well as Bac*Dive* Special Collections that the strain can be found in. **(D)** Genome-based predictions with over 90% confidence can be found integrated along experimental data in the respective sections, clearly marked with an AI icon. **(E)** All predicted data are listed in a new section along with the relevant information. **(F)** A new literature section lists publications metadata retrieved from PubMed and automatically matched to the strain via culture collection numbers and taxonomy.

Every single data point represents a physiological test, like the enzymatic conversion of a metabolite, the resistance to an antibiotic substance, spore formation, motility or Gram staining. Initially, they were stored as API test data in the database and as such were not easily searchable and accessible. Therefore, all API test data fields were carefully evaluated and for those for which equivalent data fields were available in the Bac*Dive* database, data were transformed and incorporated into the database. Thus, the data is now directly accessible side-by-side with the curated data. Unclear results were excluded during the transformation process. In total, the transformations added new datasets on enzymes, motility, hemolysis, culture media, antibiotic sensitivity, metabolite production and utilization, spore formation, and the response to Voges-Proskauer and Indole tests. To distinguish the API test data from manually curated data, every single data point is clearly marked and shows a direct link to the original API test dataset (Figure 1A). Automatically transcribed data can be hidden from the strain detail view through the ‘exclude non-curated data’ function.

What makes this data set a treasure for microbial strain research is the largely unbiased and standardized availability of the data. So far, the world of microbial strain data is dominated by comprehensive information about type strains (buried in species descriptions). As API test data are produced routinely (e.g. for quality checks) these data provide standardized information for a large number of non-type strains for which only little data would be available otherwise. The Bac*Dive* API test data set, which includes physiological data on 18 053 non-type strains, is closing an enormous knowledge gap, which is particularly important for the identification of strains for new applications.

## Linked content

Besides integrating and standardizing data, a strength of Bac*Dive* is to connect strains to related data from other resources by providing web links. On the one hand, this allows users to seamlessly explore strain data throughout different high-quality sources. On the other hand, it allows researchers to connect data via stable identifiers and generate new data sets specific to their needs. This is further facilitated by the knowledge graph described below.

Sequence information is important in many areas of microbial research, including identification, taxonomic classification, comparative and diversity analyses and phenotype–genotype comparisons. Since the beginning, enabling users to easily connect standardized phenotypic data with genomic sequence data to provide genome–phenotype inference has been of high priority to Bac*Dive*. Bac*Dive* collects accession numbers for genome assemblies and 16S rRNA genes, which are also commonly used for phylogenetic reconstructions and taxonomy, from NCBI GenBank (12), JGI IMG (13) and BV-BRC/PATRIC (14). During the past two years, the sequence data in Bac*Dive* has been cleaned up and widely extended. Metadata for all GenBank accession numbers in the database were newly downloaded and compared against the Bac*Dive* strain they were matched to, so that wrongly matched sequences could be deleted. In addition, all GenBank nucleotide accessions for contigs, chromosomes or plasmids were consolidated into the respective assemblies. Accessions for GenBank genome assemblies and 16S rRNA gene sequences are now routinely collected by going through current accession lists downloaded from the NCBI FTP server and programmatically matching them to Bac*Dive* strains via culture collection numbers and species names, also considering all synonyms known in LPSN and the NCBI taxonomy (15). Bac*Dive* now links to 50 588 genome assemblies and 41 458 16S rRNA gene sequences.

Peer-reviewed journal articles are still the most important medium for the communication of research findings. Primary literature therefore stores deep knowledge about prokaryotic strains. While we endeavor to extract standardized information from the literature, the diversity of data is too great to cover everything in a standardized form in the foreseeable future. However, the number and frequency of new publications is constantly increasing, making it very difficult to keep up with new findings. In order to provide an overview about the currently available publications related to a specific strain, we introduced a new literature table within the section ‘*External links*’ (Figure 1F). This allows researchers to easily find further material on a strain of interest. The literature is gathered automatically by collecting all species names from Bac*Dive*, downloading metadata and abstracts for all publications for this species from PubMed (16) and finally screening them for all culture collections numbers linked to Bac*Dive* strains of the species. In this way, 59 282 publications for 19 698 prokaryotic strains could be added to the database. For a better overview, the publications are categorized into one of the following topics: Biotechnology (764 publications), Cultivation (419), Enzymology (4532), Genetics (3151), Lipids (5), Metabolism (7139), Pathogenicity (6262), Phenotype (88), Phylogeny (31 007), Physiology (10), Proteome (81), Stress (223) and Transcriptome (161). To assign publications to topics, keywords are extracted from publication titles and MeSH (Medical Subject Headings) terms, excluding stop words and using a lemmatizer. The keywords are then compared against a custom dictionary for assignment to categories. The publications are linked to the original publication via Digital Object Identifier (DOI) and the metadata is available for download in BibTeX format.

For understanding the ecological role of a microbial strain, its isolation source is of major importance. Naturally, a strain is only collected once and therefore only provides a single point of evidence for the potential habitat of the species, which might not be representative. To provide a better overview about occurrences in the environment, we matched the 16S rRNA gene sequence data with data from the MicrobeAtlas database (https://microbeatlas.org/) (17). For 15 655 strains matches with at least 99% 16S rRNA gene sequence identity were found. For each strain, Taxonmaps provide a preview of the global distribution. They were integrated into the strain detail view alongside data about the environmental categorization into aquatic, soil, animal and plant (Figure 1B). By clicking on the map, users are redirected to the respective MicrobeAtlas entry providing a zoomable map, as well as further comprehensive information.

Since January 2023, Bac*Dive* is part of the newly built biodata infrastructure DSMZ Digital Diversity (https://hub.dsmz.de), which aims to establish an integrated suite of scientific databases of fundamental relevance for the life sciences. The databases not only include the established *Core data resources* BRENDA (18), SILVA (19) and LPSN (10), but also newly established databases like StrainInfo (https://straininfo.dsmz.de), Media*Dive* (20) and Phage*Dive* (21). All resources are developed in a coordinated manner and benefit from the frequent exchange of data. Stable links provide users with easy access to all resources. Linking nomenclatural data to LPSN, enzymatic data to BRENDA and 16S rRNA gene sequence data to SILVA has been established for many years already. Recently, we added links to entries for cultivation media in Media*Dive*, which provides detailed instructions for cultivation media recipes as well as comfortable functions to find alternative media or to build own media recipes. StrainInfo is a reestablished database, dedicated to strain identity information and provides additional information on the associated cultures and their relations (Figure 1C). Phage*Dive* provides comprehensive information on prokaryotic viruses that are able to infect the respective strain (Figure 1C). While linking is important, linked data are not easily queried and analyzed. Therefore, the goal of DSMZ Digital Diversity is to provide access to integrated data from multiple sources through a central hub to enable scientists to gain new insights that are currently hidden. For this reason, Bac*Dive* strains can now also be found via an integrated search through the Digital Diversity Hub (https://hub.dsmz.de/#/search/).

## Searching and querying content

As Bac*Dive* is a knowledge database, its tools are focused on searching and retrieving data. The most-frequently used tool is the *Simple Search* (starting page or top of each page) which allows users to easily find a strain based on its name, culture collection number, NCBI Tax-ID or sequence accession number. Lately this search function was supplemented with a *Smart Search*. When a user starts typing into the search bar, the *Smart Search* function makes suggestions based on almost 300 000 precalculated *Advanced Search* queries, for example recognizing search terms like ‘glucose’ and offering various search options. This bridges the gap between the *Simple Search* and the *Advanced Search* and therefore provides a low-level entry for users into the more complex search options of Bac*Dive*.

**Figure 2.**
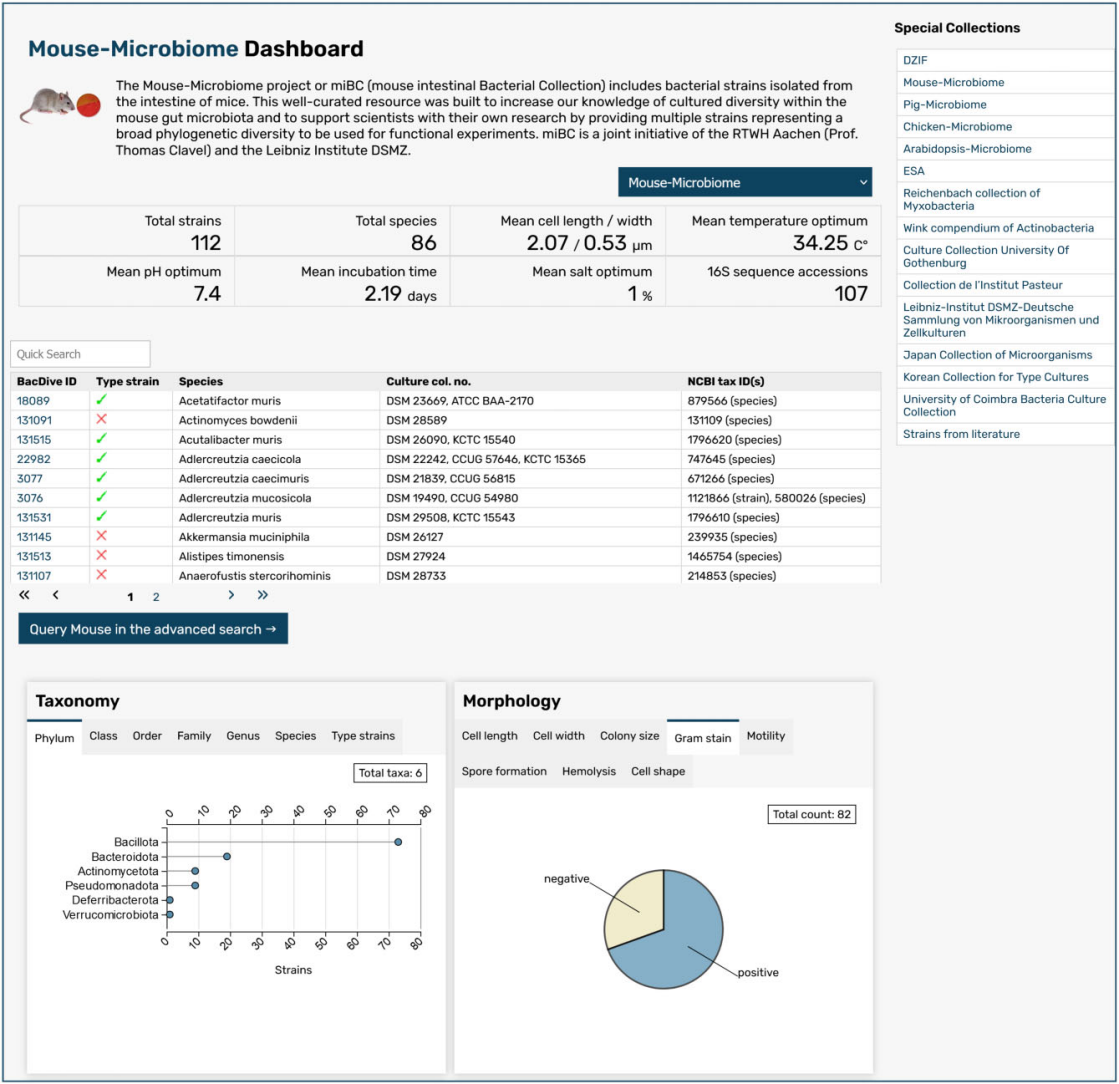
Special collection dashboard. A short description, list of strains and selected statistics give an overview of a special collection. The sidebar on the top right allows for easy switching between different collections.

Another new query tool in Bac*Dive* are so-called *Special Collections*. These group together strains that belong to specific research projects or collections, and therefore are of special interest to the user. To provide better access and visibility to these otherwise hidden data sets, these strains are displayed on dedicated *Special Collections* pages and are also query- and retrievable as a whole. An example is the Mouse Microbiome collection, or miBC (mouse intestinal Bacterial Collection), which contains strains that were isolated from the intestines of mice in a joint initiative of the RWTH Aachen and the Leibniz Institute DSMZ and deposited in the DSMZ collection to make them available for research purposes (22). Other examples are the ESA collection of strains isolated from spacecrafts and assembly facilities of the European Space Agency (ESA), and special collections containing all strains that are available through a specific Biological Resource Center. A new *Special Collections* landing page gives an overview on the available collections and provides links to the individual collection pages, where a short description and overview of the collection can be found (Figure 2).

At the top of these pages, the respective special collection is shortly described. Below, tiles contain key statistical values: the total number of strains, the total number of species, and if available, mean values for cell length and width, the cultivation temperature optimum, the cultivation pH optimum, mean incubation time, mean cultivation salt optimum and number of 16S rRNA gene accession numbers connected to the strains. These are followed by a list of all strains in the *Special Collection*, displayed as a searchable table with Bac*Dive* IDs, type strain status, species name, NCBI Taxonomy-ID, and culture collection numbers. For further exploration of the collection, a link to the Bac*Dive Advanced Search* with pre-selection for the *Special Collection* is provided. This enables users to further query the collection and easily download data for all or selected strains of the group. Additional statistics are displayed on the bottom of the page in interactively displayed graphs or charts for different aspects of taxonomy, morphology, cultivation, metabolism, isolation sources, geographical distribution, molecular biology and pathogenicity, and physiological tests. Only graphs for which data are available are displayed. Clicking on a data point in any of the graphs opens the respective search in the *Advanced Search, Isolation Source Search* or TAXplorer, which allows the user to explore the filtered data set further.

## Semantic integration of content: the Bac*Dive* knowledge graph

As a knowledge base, the mission of Bac*Dive* is to provide access to high-quality, standardized knowledge about prokaryotic strains. The tools provided to search and analyze the data are either limited in power (e.g. *Advanced Search*) or provide a full set of Bac*Dive* data (e.g. Web services) that the user still needs to query with custom tools. Here we present a knowledge graph of the Bac*Dive* database that contains >16.5 million triples and provides a powerful SPARQL endpoint to directly search and analyze the knowledge provided through Bac*Dive* in a standardized way, supported by a new descriptive ontology. Furthermore, the Bac*Dive* knowledge graph will support the integration approaches for the DSMZ Digital Diversity infrastructure previously mentioned.

The Resource Description Framework (RDF) and the RDF Query Language (SPARQL) represent foundational technologies in the realm of semantic web and linked data (23). RDF is a standard model for data interchange on the web, enabling the integration of diverse data sources with a flexible and extensible approach. SPARQL, the query language for RDF, allows for sophisticated queries directly against the data model, enabling more precise and tailored data retrieval.

The new Bac*Dive* SPARQL endpoint uses the QLever query engine (24) and can be found at https://sparql.dsmz.de/bacdive. The Bac*Dive* knowledge graph includes detailed mapping rules for so far 26 of the most critical entities, ensuring that the most relevant data is accurately represented and connected. These entities include: Strain, Reference, StrainDesignation, CultureCollectionNumber, NutritionType, GramStain, CellMotility, CellSize, CellShape, CultureMedium, CultureTemperature, CulturePH, OxygenTolerance, LocationOfOrigin, IsolationSource, EnrichmentProcedure, ColonyMorphology, SaltTolerance, SporeFormation, RiskAssessment, Pathogenicity, Enzyme, CellPigmentation, 16SSequence, GenomeSequence, GCContent. In total, the graph contains >16.5 million triples. By focusing on these entities, we have laid a robust foundation for the comprehensive integration of Bac*Dive* data into broader knowledge frameworks, which can be further expanded in the future.

**Figure 3.**
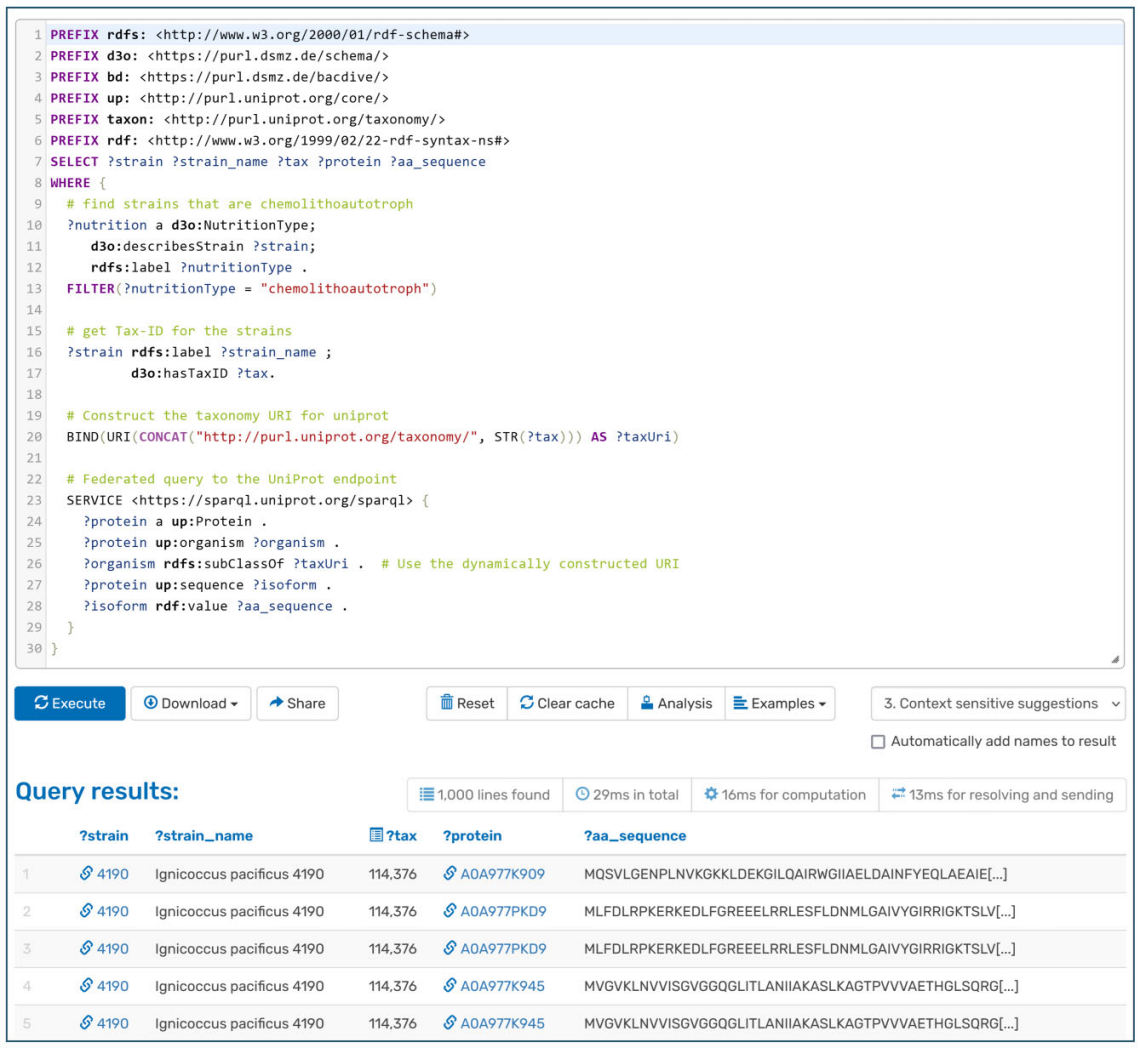
Interface to the SPARQL end point with an example of a federated SPARQL query executed on the Bac*Dive* database. The query dynamically constructs URLs for taxonomy IDs from Bac*Dive* to fetch relevant protein data, showcasing the integration of microbiological and protein sequence data through a federated query approach. The results display strains, their names, taxonomy IDs, associated proteins, and partial amino acid sequences, demonstrating the powerful capability of federated queries in combining data from disparate sources. The visible result is limited to 1000 entries, but the full set can be downloaded.

This extensive RDF dataset not only supports complex queries via SPARQL but also enables federated queries (25), which span multiple data sources. For example, microbial data from Bac*Dive* can be seamlessly integrated with protein information from UniProt (26). The potential to conduct such federated queries greatly expands the scope of scientific inquiry, allowing researchers to derive insights from a more holistic dataset than would be possible from isolated sources. A demonstration of this functionality is provided in a sample query, which retrieves all chemolithoautotrophic strains from Bac*Dive* along with their corresponding protein sequences from UniProt (Figure 3). A key point of integration is the NCBI Taxonomy-ID, directly linked to Bac*Dive* strains with the *hasTaxID* predicate. To ensure compatibility with other DSMZ databases, the knowledge graph adheres to the DSMZ Digital Diversity Ontology (D3O; https://bioportal.bioontology.org/ontologies/D3O). This allows users to query Bac*Dive* data effortlessly and integrate it with other DSMZ endpoints, such as Media*Dive*.

We have included a variety of example SPARQL queries that demonstrate the powerful capabilities of querying the Bac*Dive* knowledge graphs. These will be continually expanded with more complex and varied queries to cater to the diverse needs of researchers. The deployment of the Bac*Dive* knowledge graph with its SPARQL endpoint is an important step towards more interconnected microbiological datasets.

## Filling content gaps

While the number of data points in Bac*Dive* rises constantly, this is mainly due to the integration of new strains. Data availability for each strain varies widely and unfortunately large gaps remain in the phenotypic descriptions of many strains. This even concerns such fundamental information as the Gram staining behavior or oxygen tolerance of the strain. In order to decrease some of these profound gaps, we have recently introduced genome-based predictions from two different projects, DiASPora (https://diaspora-project.de) and deepG (https://deepg.de/), into Bac*Dive*.

The DiASPora procedure for producing strain-level phenotype predictions using public genome sequences and high-quality standardized data from Bac*Dive* is described in detail by Koblitz *et al.* (27). In short, machine learning models for several traits were trained on curated Bac*Dive* data and Pfam (28) annotated genomes using the Random Forest algorithm (29). Six models performed well enough to be used to generate data for integration into the database. For 15 938 strains with high-quality genome assemblies, new data points could be predicted for flagellated motility, Gram stain, oxygen tolerance (models for aerobe and anaerobe growth), spore-formation and growth temperature range (prediction of thermophilic growth). A second set of machine learning data was integrated into Bac*Dive* that was created using a deep learning model trained using the deepG platform (30). It provides predictions for the spore-formation ability of 9023 strains.

**Figure 4.**
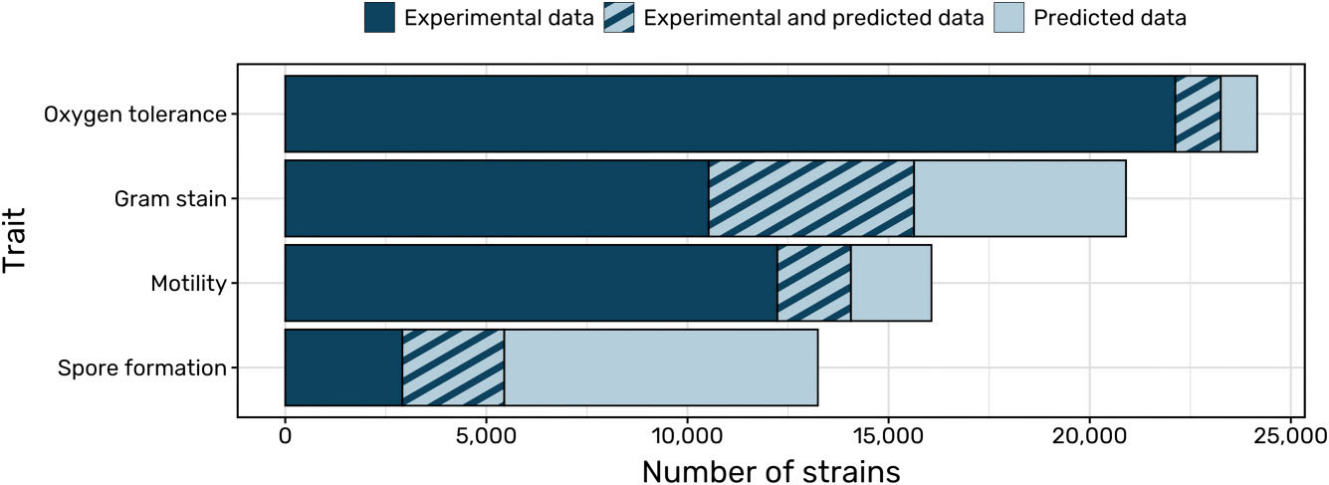
Data points added by predictions. Number of Bac*Dive* strains with data for the traits oxygen tolerance, Gram stain, motility and spore formation. Dark blue sections show numbers of strains for which only experimental values are present in the database, striped sections visualize strains for which predictions were integrated next to existing experimental data and light blue sections represent strains for which integrated predictions added information where none was previously present.

All predicted data are listed in a new section on the strain detail page titled ‘*Genome-based predictions*’ with confidence values and the link to the genome the predictions are based on. Predicted data points with over 90% confidence are additionally integrated into the other sections alongside experimental data, marked with their confidence value and an AI icon. Overall, 104 651 data points were generated by genome-based predictions covering 16 131 strains. 15 977 of these data points have added completely new information where no experimental data is available (Figure 4).

With 16 131 strains for which prediction data is available, only 17% of the strains in Bac*Dive* are covered. Nevertheless, 12 718 strains represent type strains, and thereby 62% of the currently validly described prokaryotic species are represented. The major limitation is the availability of high-quality genomes for less well-described strains. With the progress in sequencing technology, the importance of interpreting genome functions by machine learning models will rise over the next years. Bac*Dive* is an ideal platform to integrate data resulting from genome-based predictions, as high-quality predictions can fill knowledge gaps for not well-described strains and challenge existing data to improve the data quality. Moreover, by applying published models and integrating the data generated by them, Bac*Dive* offers a great way to make them more findable, accessible, interoperable, and reusable (FAIR).

## Data Availability

Bac*Dive* data can be freely downloaded in various formats (e.g. CSV, JSON, PDF) without restrictions, except that the origin of the data has to be properly cited when used in other works (CC BY 4.0 license). Registration is necessary to access data through the RESTful API, but registration is free of charge. The AI models from the DiASPora project can be found at https://doi.org/10.5281/zenodo.13757323.
